# Pulmonary Toxicities of Gefitinib in Patients With Advanced Non-Small-Cell Lung Cancer: A Meta-Analysis of Randomized Controlled Trials

**DOI:** 10.1097/MD.0000000000003008

**Published:** 2016-03-07

**Authors:** Dongsheng Hong, Guobing Zhang, Xingguo Zhang, Xingguang Lian

**Affiliations:** From the Department of Pharmacy (DH, GZ, XZ), The First Affiliated Hospital of College of Medicine, Zhejiang University; and Central Laboratory (XL), The First Affiliated Hospital of College of Medicine, Zhejiang University, Hangzhou, China.

## Abstract

Gefitinib is a selective tyrosine kinase inhibitor of the epidermal growth factor receptor (EGFR) used to treat adults with EGFR mutation-positive non-small-cell lung cancer (NSCLC). Clinical benefits of gefitinib administration in NSCLC patients have been observed in clinical practice, but the extent of the pulmonary toxicity of gefitinib in patients with advanced NSCLC remains unclear.

The aim of this systematic review was to evaluate the overall incidence and risk of gefitinib-related pulmonary toxicity in advanced NSCLC patients.

Relevant trials were identified from the databases of Pubmed, Embase, Cochrane Library, and the clinicaltrials.gov of the U.S. National Institutes of Health. The outcomes included the overall incidence, odds ratios (ORs), and 95% confidence intervals (CIs). Fixed-effects models were used in the statistical analyses according to the heterogeneity of the included studies.

According to the data from the included trials, the overall incidence of high-grade hemoptysis, pneumonia, pneumonitis, and interstitial lung disease (ILD) was 0.49% (95% CI: 0.24%–0.99%), 2.33% (95% CI: 1.47%–3.66%), 2.24% (95% CI: 1.34%–3.72%), and 1.43% (95% CI: 0.98%–2.09%), respectively. The pooled ORs of high-grade hemoptysis, pneumonia, pneumonitis, and ILD were 1.73 (95% CI: 0.46–6.52; *P* = 0.42), 0.99 (95% CI: 0.66–1.49; *P* = 0.95), 4.70 (95% CI: 1.48–14.95; *P *= 0.0087), and 2.64 (95% CI: 1.22–5.69; *P* = 0.01), respectively.

Gefitinib was associated with a significantly increased risk of high-grade/fatal ILD and pneumonitis compared with the controls, whereas the risk of other high-grade pulmonary events (pneumonia and hemoptysis) was not significant. Careful surveillance of gefitinib-related pulmonary toxicity is critical for the safe use of this drug.

## Abstract 2

Supplemental Digital Content is available in the text

## INTRODUCTION

Gefitinib is a selective tyrosine kinase inhibitor (TKI) of the epidermal growth factor receptor (EGFR) used to treat adults with EGFR mutation-positive non-small-cell lung cancer (NSCLC).^1,2^ Clinical benefits of gefitinib administration in NSCLC patients have been observed in clinical practice.^3^ However, gefitinib is also associated with adverse events such as diarrhea, skin rash, and stomatitis.^4,5^ In addition, clinical trials have reported a broad range of gefitinib-related adverse pulmonary events, including interstitial lung disease (ILD) and pneumonia.^6^ The high incidence of adverse events is due to the characteristics and action mechanisms of gefitinib, which differs from those of conventional chemotherapy agents.^7^ Pulmonary toxicity has a poor prognosis because its pathophysiology is unclear, and therapeutic options are limited. Thus, the incidence of gefitinib-related pulmonary toxicity must be understood.

Recently, gefitinib was shown to be more effective than placebo in increasing overall survival, and gefitinib was approved for NSCLC patients after the failure of chemotherapy.^8,9^ However, clinical trials have reported substantial variations in the incidence of pulmonary toxicity events,^10–12^ and death caused by pulmonary toxicity has been reported. Based on difficulties in interpreting and analyzing data from randomized controlled trials (RCTs), the extent of the pulmonary toxicity of gefitinib in patients with advanced NSCLC remains unclear. Furthermore, poor management of pulmonary toxicity may lead to serious lung injury or have life-threatening consequences. Therefore, this meta-analysis was conducted to investigate the pulmonary toxicity of gefitinib in advanced NSCLC patients.

## METHODS

### Search Strategy and Study Selection

This study was a meta-analysis and did not involve human subjects, and therefore ethical approval was not required. Searches were conducted of the electronic databases Embase from 1974, PubMed from 1967, and the Cochrane Library to the end of August 2015. The following search terms, treated as free text or mesh terms, were used: “non-small-cell lung cancer,” “carcinoma,” “non-small-cell lung,” “gefitinib,” “randomized controlled trials,” “clinical trials,” “controlled clinical trials,” “clinical trial as topic,” or “randomized controlled trial as topic.” For details of the search strategy, please see Supplemental Content. Abstracts and virtual presentations from the American Society of Clinical Oncology (http://www.asco.org/ASCO) up to 2015 were also searched for relevant RCTs. In addition, the clinicaltrials.gov of the U.S. National Institutes of Health was searched.

The selection of studies was conducted according to the Preferred Reporting Items for Systematic Reviews and Meta-Analyses (PRISMA) statement. Clinical trials that met the following criteria were included:

1. Phase II, III, and IV trials in patients with advanced NSCLC.
2. Participants who received daily gefitinib treatment.
3. Events or event rates and sample sizes available for hemoptysis, pneumonia, pneumonitis, and ILD.

Data on the pulmonary toxicity were extracted from the safety profile of each RCT. The clinical endpoints were classified according to the Common Terminology Criteria for Adverse Events of the National Cancer Institute.

### Data Extraction and Quality Assessment

Two reviewers (DH and GZ) independently extracted the data from the included trials. In cases of a lack of agreement by the 2 reviewers, the data were subjected to another review until a consensus was reached. The following information was collected for each trial: first investigator's name, year of publication, study type, treatment arm, number of patients in the gefitinib and control groups, and number of high-grade pulmonary toxicity events. The quality of the methodologies in each trials was assessed using the Jadad criteria.^13^ The quality of each included trials was graded, with high-quality trials classified as those with a score of ≥3 and low-quality trials classified as those with a score of ≤2.

### Data Analysis

Data on patients with pulmonary toxicity and on patients treated with gefitinib were extracted from the safety profiles of all the included trials, and the proportions and 95% confidence intervals (CIs) of the patients with pulmonary toxicity were calculated to access the incidence. For trials with a control group, the odds ratios (ORs) of pulmonary toxicity were also calculated. A statistical test was considered significant if the *P* value was <0.05. Peto's method was used to calculate the ORs and 95% CIs. This method specifies the best reliance interval coverage, and it is more convincing with less bias while to process low event rates relatively.^14^ The heterogeneity assumption was assessed by the Q statistic and I^2^ tests.^15,16^ A *P* value of <0.1 or I^2^ > 40% indicated statistically significant heterogeneity, and the random-effects model was used. A fixed-effects model was used when heterogeneity did not exist. Potential publication bias was estimated by Egger tests, Begg test, and the funnel plot.^17,18^ All the meta-analyses were conducted with R software, version 3.0.3 (The R Foundation for Statistical Computing, http://www.r-project.org).

## RESULTS

### Search Results and Characteristics of the Patients

**FIGURE 1:**
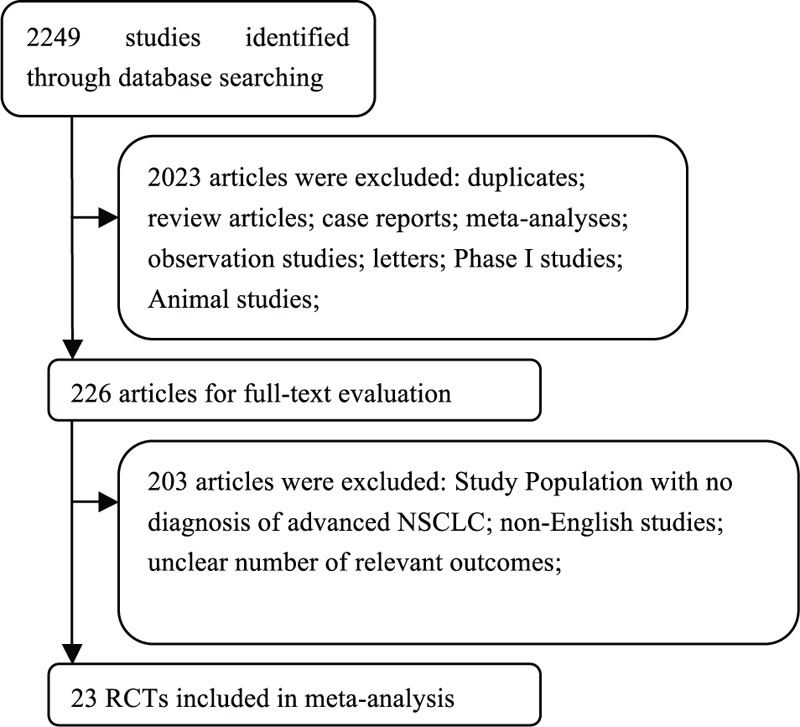
Flow chart demonstrating the process of study selection.

A total of 2249 potentially relevant citations were retrieved from the initial search. Twenty-three studies,^19–41^ including 9054 subjects, met the inclusion criteria in the search strategy and study selection. Figure 1 outlines the selection process in detail. Of the included studies, 7 were phase II RCTs,^19–25^ 15 were phase III RCTs,^26–40^ and 1 was a phase IV RCT.^41^ The sample sizes ranged from 16 to 1126 subjects (median sample size, 150 subjects). All the included studies illustrated appropriate methods of blinding and randomization, and the Jadad scores ranged from 3 to 5. According to the inclusion criteria, subjects with abnormal bone marrow function and hepatic or renal dysfunction were not included. Table 1 presents the data on high-grade/fatal events and major baseline characteristics of the 23 studies.

**TABLE 1:**
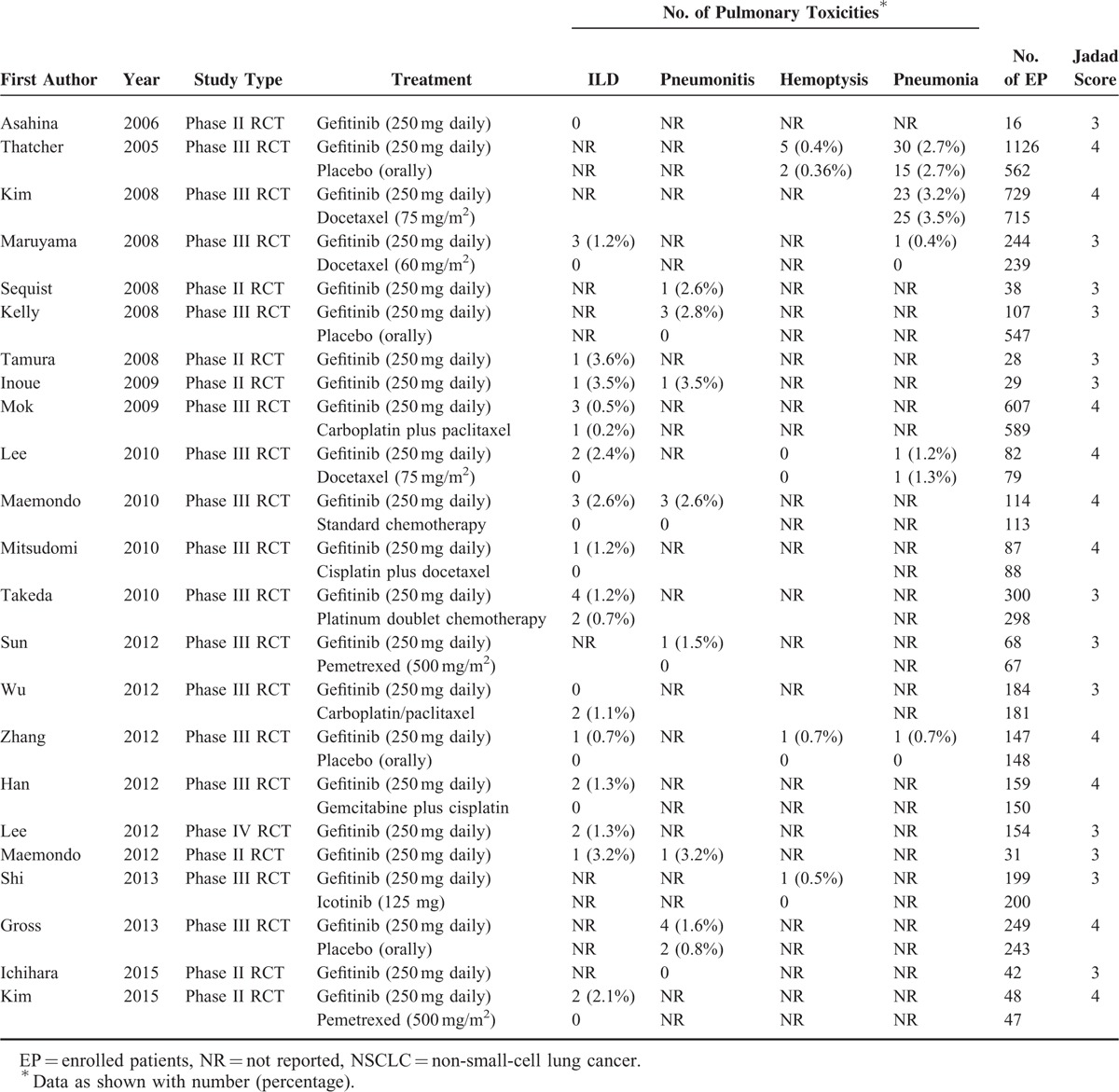
Fatal or High-Grade Pulmonary Toxicities of Gefitinib in Patients With NSCLC in Our Study (n = 9054)

The guidelines of the PRISMA statement were followed in this meta-analysis (see the Guidelines Checklist).

### Overall Incidence of High-Grade Pulmonary Toxicity

The incidence of high-grade pulmonary toxicity in 4472 subjects treated with gefitinib were analyzed in 23 studies. The index of pulmonary toxicity mainly included ILD, pneumonitis, pneumonia, and hemoptysis. High-grade ILD was observed in 15 of the 23 studies, and 25 events were detected among these patients. The incidence of high-grade ILD was between 0% and 3.57%. High-grade pneumonitis was observed in 8 of the 23 studies, and 14 total events occurred among these patients. The incidence of high-grade pneumonitis was between 0% and 3.45%. High-grade pneumonia was observed in 5 of the 23 studies, and 56 events occurred among these patients. The incidence of high-grade pneumonia was between 0.41% and 3.16%. High-grade hemoptysis was observed in 4 of the 23 studies, with 7 events among these patients. The incidence of high-grade hemoptysis was between 0% and 0.68%.

**FIGURE 2:**
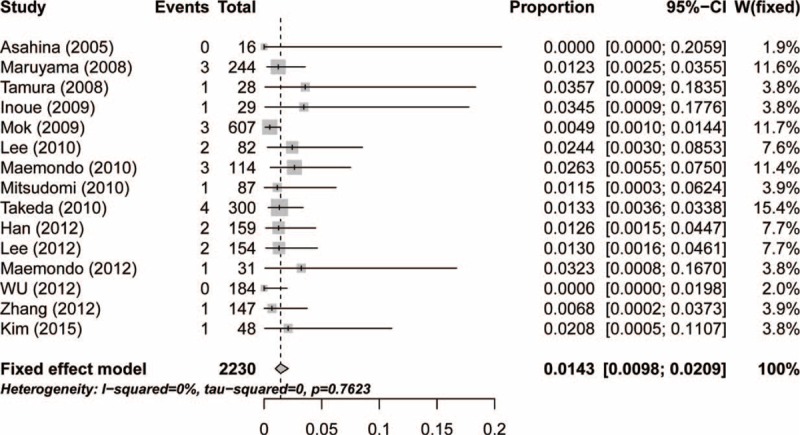
Forest plot for incidence of interstitial lung disease in patients with advanced non-small-cell lung cancer.

According to the data from the included trials, the overall incidence of high-grade hemoptysis, pneumonia, pneumonitis, and ILD was 0.49% (95% CI: 0.24%–0.99%; Table 2), 2.33% (95% CI: 1.47%–3.66%; Table 2), 2.24% (95% CI: 1.34%–3.72%; Table 2), and 1.43% (95% CI: 0.98%–2.09%; Figure 2), respectively, according to the fixed-effects model.

**TABLE 2:**
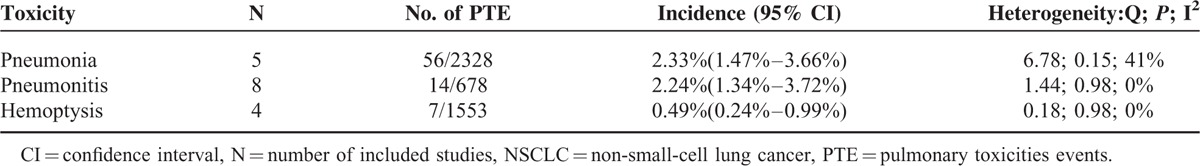
Overall Incidences of Other Pulmonary Toxicities in Patients With Advanced NSCLC Assigned to Gefitinib

### Relative Risk of High-Grade Pulmonary Toxicity

**FIGURE 3:**
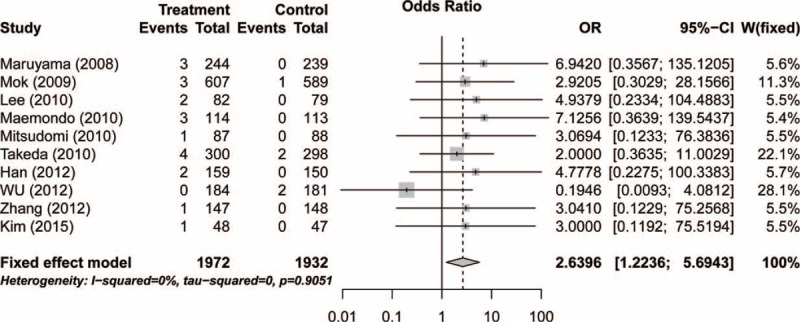
Relative risk of associated interstitial lung disease with gefitinib versus controls.

To determine the specific contribution of gefitinib to the development of pulmonary toxicity, excluding the influence of confounding factors, such as a history of other therapeutic interventions, we calculated the ORs of high-grade pulmonary toxicity (hemoptysis, pneumonia, pneumonitis, and ILD) in the gefitinib and control groups in 16 RCTs. The pooled ORs of high-grade hemoptysis, pneumonia, pneumonitis and ILD were 1.73 (95% CI: 0.46–6.52; *P* = 0.42; Table 3), 0.99 (95% CI: 0.66–1.49; *P* = 0.95; Table 3), 4.70 (95% CI: 1.48–14.95; *P* = 0.0087; Table 3), and 2.64 (95% CI: 1.22–5.69; *P* = 0.01; Figure 3), respectively, according to the fixed-effects model. The results indicated that patients who received gefitinib had a significantly increased risk of high-grade ILD and pneumonitis.

**TABLE 3:**
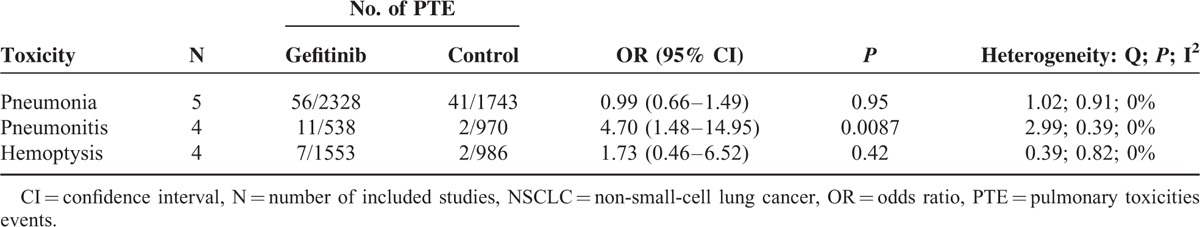
Meta-Analysis of Other Pulmonary Toxicities in Patients With Advanced NSCLC Assigned to Gefitinib or Control Intervention

### Publication Bias

**FIGURE 4:**
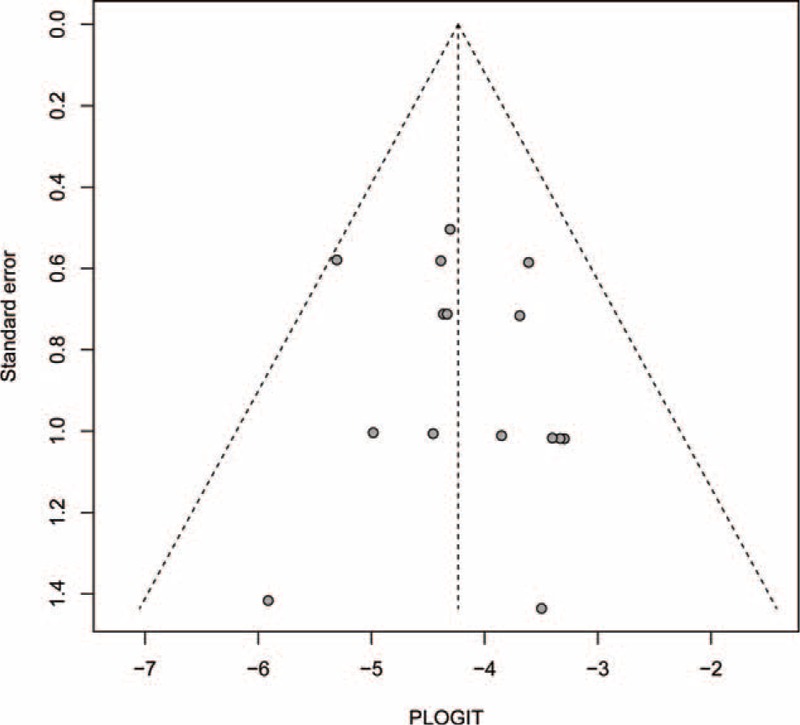
Funnel plot standard error by odds ratio for interstitial lung disease.

No evidence of publication bias was found for the OR of high-grade pulmonary toxicity in the meta-analysis according to the funnel plots (Figure 4), Egger test (*P* = 0.656 > 0.05, 95% CI: −1.82, 2.73), or Begg test (Z = 0.858 < 1.96, *P *= 0.656 > 0.05).

## DISCUSSION

The management and diagnosis of pulmonary toxicity in patients with NSCLC is problematic for a number of reasons. First, it can be difficult to delineate pulmonary toxicity-related adverse events from disease progression.^42^ Second, dose decreases and/or discontinued treatment may reduce pulmonary toxicity but not yield cancer control. Although pulmonary toxicity has been reported in clinical trials of NSCLC patients treated with gefitinib,^19,29^ no systematic attempt has been made to synthesize the available data. Thus, the aim of this meta-analysis was to evaluate the pulmonary toxicity of gefitinib in patients with advanced NSCLC.

A meta-analysis is a formidable statistical tool, which can be used to assess the incidence and risk factors of drug-related adverse reactions. The results of a meta-analysis can increase the number of clinical samples and the statistical evidence can improve. In addition, conclusions based on meta-analyses are more valid than those when selecting suitable therapeutic schemes in clinical practice. This meta-analysis is the latest study to evaluate the wide spectrum of the incidence of pulmonary toxicity-related adverse events in NSCLC patients treated with gefitinib. This meta-analysis included 9054 subjects from 23 studies. The findings demonstrated that the overall incidences of high-grade hemoptysis, pneumonia, pneumonitis, and ILD were 0.49%, 2.33%, 2.24%, and 1.43%, respectively. In addition, the use of gefitinib was associated with a significantly increased incidence of high-grade ILD and pneumonitis compared with the controls, whereas the risk of high-grade pneumonia and fatal hemoptysis was insignificant. The ORs of high-grade hemoptysis, pneumonia, pneumonitis, and ILD were 1.73, 0.99, 4.70, and 2.64, respectively. Therefore, gefitinib treatment was associated with an unexpectedly high risk of developing pulmonary toxicity, especially ILD and pneumonitis. Continuous monitoring and effective preventive management are crucial during gefitinib treatment.

Gefitinib is an orally active EGFR-TKI, which is used extensively to treat advanced NSCLC. In practice, it is used to treat a more heterogeneous patient population than in clinical trials. Much effort is required to limit the risk of pulmonary toxicity. The above adverse events of pulmonary toxicity likely contribute significantly to changes or discontinuation of treatment schedules in clinical trials.^31,32^ Thus, routine monitoring of patients receiving gefitinib is necessary. It may not be possible to differentiate between pulmonary toxicity-related adverse events and disease progression in patients with NSCLS, even after radiological and extensive clinical. Therefore, all NSCLC patients receiving should be cautioned and carefully care if they experience any suspected adverse events of pulmonary toxicity.

The molecular mechanism of gefitinib-related pulmonary toxicity is currently unknown, and it is possible that multiple factors are involved. Idiopathic pulmonary fibrosis (IPF) is the most common form of pulmonary toxicity. Lysophosphatidic acid (LPA), a multifunctional phospholipid, plays an important role in the pathogenesis of IPF and leads to fibroblast growth, proliferation, and migration.^44^ A previous study reported that the concentration of LPA in broncho-alveolar lavage (BAL) fluid of patients with IPF was clearly, obviously higher than that of healthy controls.^45^ In a bleomycin mouse model of pulmonary fibrosis, the concentration of LPA in BAL fluid markedly increased after 5 days,^45^ and LPA1-deficient mice showed significant resistance to bleomycin-induced IPF. An LPA receptor antagonist has been approved for the inhibition of lung fibrosis. The overexpression of β-catenin was also found to be associated with gefitinib resistance in NSCLC,^46^ and suppressing the activity of Wnt signaling partly reversed cellular resistance to gefitinib in NSCLC.^47^

Gefitinib acts clinically as an EGFR inhibitor. However, its side effects on other lung tissue are a concern. Targeted delivery could be a solution to this problem. In a previous study, receptor tyrosine kinase-like orphan receptor 1 (ROR1) bound to EGFR led to activation of the phosphatidylinositol 3 kinase (PI3K) pathway, which is critical in lung cancer. Interestingly, surface ROR1 has been found to be a good target for cancer treatment. A recent study described a novel strategy to deliver toxic reagents, specifically by targeting ROR1.^48,49^

The downstream signaling transduction pathways of ROR1 are confusing, but a recent study, which focused on mAb and the hsRNA inhibitor ROR1, suggested that the PI3K/AKT/mTOR pathway might be involved.^50^ The findings of that study indicate that a cell survival signaling pathway, mediated by ROR1, may exist in NSCLC.

The etiology of gefitinib-related pulmonary toxicity is complex. Although early studies did not detect specific gefitinib-related pulmonary toxicity, large multiple studies have since emphasized its association with pulmonary fibrosis. One report suggested that blocking the expression of heat shock protein 70 seemed to exacerbate gefitinib-induced pulmonary fibrosis.^51^ Therefore, an inducer of heat shock protein 70 expression, such as geranylgeranylacetone, may be clinically useful in the treatment of pulmonary fibrosis induced by gefitinib. Pulmonary fibrosis is produced by repeated damage of epithelial cells caused by reactive oxygen species and other stressors, with abnormal wound remodeling and repair leading to abnormal deposition of extracellular matrix proteins, such as collagen. Previous studies suggested that elevated levels of transforming growth factors,^52^ which could increase the numbers of lung myofibroblasts, might play a critical role in pulmonary fibrosis^42^ and that oxidative stress may act as a causative agent in the exacerbation of gefitinib-induced ILD.^53^

Clinical manifestations of gefitinib-induced pulmonary toxicity include an unproductive cough, dyspnea, and serious hypoxemia.^43,54^ A definitive diagnosis is based on clinical symptoms, characteristic findings of a computed tomography scan, nonprogression of NSCLC, and a curative effect upon cessation of gefitinib.^6,43^ Therefore, it is often difficult to obtain an accurate diagnosis of gefitinib-induced pulmonary toxicity. The appearance of respiratory symptoms within the first 4 weeks of gefitinib treatment can provide important evidence and aid the early diagnosis of gefitinib-induced pulmonary toxicity.^55^ Chest CT is also useful in detecting. Until now, there have been no RCTs of the factors underlying gefitinib-induced pulmonary toxicity. Current recommendations for such toxicity are immediate discontinuation of gefitinib, oxygen therapy, and intravenous corticosteroids.^43^ Successful therapy with large-dose corticosteroids has been reported for gefitinib-induced pulmonary toxicity intractable to a medium dosage of corticosteroids.^56^

In the present meta-analysis, several limitations need to be considered. First, the studies included in the meta-analysis were conducted at various international institutions by different investigators and may have bias in reporting adverse events. In particular, the frequency of pulmonary toxicity is underreported in clinical trials. Second, RCTs have strict inclusion and exclusion criteria. Only patients with adequate major organ function were included in these trials. Therefore, the results of the meta-analysis may not represent those found in patients.^57,58^ Third, the baseline pulmonary toxicity of the patients was not described in the included trials, which may have led to an overestimation of the incidence of gefitinib-induced pulmonary toxicity.

## CONCLUSION

In conclusion, this meta-analysis showed that gefitinib was associated with a significantly increased risk of high-grade/fatal ILD and pneumonitis compared with the controls, whereas the risk of other high-grade pulmonary events (pneumonia and hemoptysis) was not significant. Surveillance of adverse events is critical under gefitinib treatment to detect pulmonary toxicity. Early diagnosis and management, including discontinuing gefitinib and/or treatment with intravenous corticosteroids, may avert a fatal outcome. Careful surveillance of pulmonary toxicity is critical for safer drug use.

## Supplementary Material

Supplemental Digital Content
