# Pulmonary Toxicities of Gefitinib in Patients With Advanced Non-Small-Cell Lung Cancer: A Meta-Analysis of Randomized Controlled Trials: Erratum

**DOI:** 10.1097/01.md.0000490127.42415.6a

**Published:** 2016-07-29

In the article “Pulmonary Toxicities of Gefitinib in Patients With Advanced Non-Small-Cell Lung Cancer: A Meta-Analysis of Randomized Controlled Trials”,^1^ which appeared in Volume 95, Issue 9 of *Medicine*, Dr. Liang's name was incorrectly spelled as Lian. The article has since been corrected online.
