# Supplementary material for: Pulmonary Toxicities of Gefitinib in Patients With Advanced Non-Small-Cell Lung Cancer: A Meta-Analysis of Randomized Controlled Trials
Source: Medicine (Baltimore). 2016 Mar 7;95(9):e3008. doi: 10.1097/MD.0000000000003008 (PMC4782910; doi:10.1097/MD.0000000000003008)
Supplement: Supplemental Digital Content [file medi-95-e3008-s001.doc]

**Cochrane Library search strategy：**

1、Gefitinib:ti,ab,kw (Word variations have been searched)；

2、Randomized controlled trials:ti,ab,kw (Word variations have been searched)

3、Clinical trials:ti,ab,kw (Word variations have been searched)

4、non-small-cell lung cancer:ti,ab,kw (Word variations have been searched)

5、MeSH descriptor: [Carcinoma, Non-Small-Cell Lung] explode all trees

6、MeSH descriptor: [Randomized Controlled Trial] explode all trees

7、MeSH descriptor: [Clinical Trial] explode all trees

8、MeSH descriptor: [Controlled Clinical Trial] explode all trees

9、Controlled clinical trials:ti,ab,kw (Word variations have been searched)

10、#2 or #3 or #6 or #7 or #8 or #9

11、#4 or #5

12、#1 and #10 and #11

**Pubmed search strategy：**

1、Gefitinib[Title/Abstract]；

2、gefitinib" [Supplementary Concept]；

3、Randomized controlled trials[Title/Abstract]；

4、Randomized Controlled Trials as Topic"[Mesh]；

5、Clinical trials[Title/Abstract]；

6、Clinical Trials as Topic"[Mesh]；

7、Controlled clinical trials[Title/Abstract]；

8、Controlled Clinical Trials as Topic"[Mesh]；

9、non-small-cell lung cancer[Title/Abstract]；

10、"Carcinoma, Non-Small-Cell Lung"[Mesh]；

11、Search (Gefitinib[Title/Abstract]) OR "gefitinib" [Supplementary Concept]；

12、Search (((((Randomized controlled trials[Title/Abstract]) OR "Randomized Controlled Trials as Topic"[Mesh]) OR Clinical trials[Title/Abstract]) OR "Clinical Trials as Topic"[Mesh]) OR Controlled clinical trials[Title/Abstract]) OR "Controlled Clinical Trials as Topic"[Mesh]；

13、Search (non-small-cell lung cancer[Title/Abstract]) OR "Carcinoma, Non-Small-Cell Lung"[Mesh]；

14、Search ((((Gefitinib[Title/Abstract]) OR "gefitinib" [Supplementary Concept])) AND ((((((Randomized controlled trials[Title/Abstract]) OR "Randomized Controlled Trials as Topic"[Mesh]) OR Clinical trials[Title/Abstract]) OR "Clinical Trials as Topic"[Mesh]) OR Controlled clinical trials[Title/Abstract]) OR "Controlled Clinical Trials as Topic"[Mesh])) AND ((non-small-cell lung cancer[Title/Abstract]) OR "Carcinoma, Non-Small-Cell Lung"[Mesh])

**Embase search strategy：**

1、'gefitinib':ab,ti；

2、'gefitinib'/exp；

3、'randomized controlled trials':ab,ti；

4、'clinical trials':ab,ti；

5、'controlled clinical trials':ab,ti；

6、'non-small-cell lung cancer':ab,ti；

7、'non small cell lung cancer'/exp；

8、'clinical trial (topic)'/exp；

9、'controlled clinical trial (topic)'/exp；

10、'randomized controlled trial (topic)'/exp

11、#1 OR #2

12、#3 OR #4 OR #5 OR #8 OR #9 OR #11

13、#6 OR #7

14、#12 AND #13 AND #14
